# Evaluation of non-invasive diagnostic tests for *Mycoplasma pneumoniae* pneumonia

**DOI:** 10.3389/fimmu.2025.1656192

**Published:** 2025-09-30

**Authors:** Yongwei Fan, Zhenyun Tan, Zhenfa Wang, Huaqin Pan, Jingrun Zhou, Jiong Yang, Guqin Zhang

**Affiliations:** ^1^ Department of Respiratory and Critical Care Medicine, Zhongnan Hospital of Wuhan University, Wuhan, China; ^2^ Institute of Hepatobiliary Diseases and Transplantation ICU, Zhongnan Hospital of Wuhan University, Wuhan, China; ^3^ Department of Critical Care Medicine, Zhongnan Hospital of Wuhan University, Clinical Research Center of Hubei Critical Care Medicine, Wuhan, China; ^4^ Respiratory Department, Second Affiliated Hospital of Hainan Medical University, Haikou, China

**Keywords:** *Mycoplasma pneumoniae*, diagnostic accuracy, nasopharyngeal swab nucleic acid testing, IgM antibody, metagenomic next-generation sequencing, community-acquired pneumonia

## Abstract

**Background:**

Accurate and timely identification of *Mycoplasma pneumoniae* pneumonia (MPP) remains a clinical challenge. Although nasopharyngeal swab nucleic acid testing (NAAT) and serum IgM antibody assays are widely used, their diagnostic performance varies across studies. This study aimed to retrospectively evaluate the sensitivity and specificity of the two non-invasive methods (NAAT and serum IgM antibody assays) for MPP in real-world clinical settings.

**Methods:**

We conducted a retrospective study of adult patients hospitalized for community-acquired pneumonia (CAP) from January 2024 to October 2024. All enrolled patients underwent bronchoalveolar lavage fluid metagenomic next-generation sequencing (BALF-mNGS) and had received at least one of two non-invasive tests (NAAT or serum IgM antibody assays). The sensitivity and specificity of NAAT and serum IgM antibody assays were calculated against the final diagnosis. A non-inferiority test was used to determine whether the sensitivity of NAAT or serum IgM antibody assays was not inferior to that of mNGS.

**Results:**

Among 594 patients included in the analysis, 60 were diagnosed with MPP based on a composite reference standard that included laboratory testing results and adjudication by two senior clinicians in accordance with clinical and radiological findings. The sensitivity and specificity of NAAT were 74.1% and 99.3%, respectively, while those of serum IgM antibody assays were 23.6% and 98.0%. McNemar’s test revealed a statistically significant difference in sensitivity between mNGS and the two non-invasive tests (NAAT and serum IgM antibody assays) (*P*<0.05). The non-inferiority analysis revealed that both NAAT (sensitivity difference: -24.2%, 95% CI: -36.1 to -12.1%; *P*<0.01) and serum IgM antibody assays (-76.5%, 95% CI: -96.6 to -56.3%; *P*<0.01) failed to meet the 10% non-inferiority margin compared to mNGS.

**Conclusion:**

In clinical practice, a positive result from either NAAT or serum IgM antibody assays can serve as reliable adjunct evidence for diagnosing MPP. However, in cases with a high clinical suspicion of MPP, negative results from both methods are not sufficient to rule out the diagnosis. For MPP, mNGS remains the most effective diagnostic method compared to non-invasive testing alternatives.

## 1 Introduction

Among the pathogens responsible for community-acquired pneumonia (CAP) in adults, *Mycoplasma pneumoniae* (*M. pneumoniae*) occupies a significant position (1, 2). A systematic analysis from the 2016 Global Burden of Disease Study indicated that *M. pneumoniae* and *Streptococcus pneumoniae* are the predominant pathogens causing CAP in the U.S. population (3). In addition, CAP caused by *M. pneumoniae* can account for approximately 20–40% of total cases during pneumonia epidemics (4, 5). Similar trends have been observed in China. A multicenter prospective study reported that the prevalence of *M. pneumoniae* has exceeded that of *Streptococcus pneumoniae* among patients diagnosed with CAP in China (6, 7). Moreover, *M. pneumoniae* can elicit a broad spectrum of clinical manifestations ranging from mild upper respiratory tract infections to *M. pneumoniae* pneumonia (MPP) complicated by respiratory failure through its distinctive adhesion proteins and immune evasion mechanisms (such as inflammation driven by CARDS toxin) (8–11). These infections may trigger extrapulmonary complications affecting the skin, musculoskeletal and central nervous systems, which can be life-threatening in severe cases (12).

The 2019 guidelines from the American Thoracic Society (ATS) and the Infectious Diseases Society of America (IDSA) recommend penicillins or cephalosporins as first-line therapy for CAP patients without chronic comorbidities or risk factors (13). Similar recommendations are included in the 2016 edition of the Chinese guidelines for the diagnosis and treatment of CAP in adults (14). However, *M. pneumoniae* is intrinsically resistant to penicillins and cephalosporins, due to the absence of a cell wall. Therefore, early identification of MPP and timely initiation of targeted antibiotic therapy are essential to improve clinical outcomes (12, 14, 15).

Currently, clinical laboratory diagnosis of MPP relies on three main approaches: culture, serology, and molecular techniques. According to the 2016 Chinese guidelines, two methods are accepted as definitive diagnostic criteria for MPP (14). First, positive culture results from qualified respiratory specimens (e.g., sputum or alveolar lavage fluid, etc.) provide direct evidence of *M. pneumoniae* infection and support an MPP diagnosis (14). However, the practical utility of culture is limited by the prolonged growth cycle of *M. pneumoniae* and a low positive culture rate, despite advances in culture techniques (16–18). Second, serological diagnosis is based on detecting a fourfold or greater change in *M. pneumoniae*-specific antibody titers between the acute and convalescent phases, which confirms infection (14). However, delayed antibody response restricts the value of serology for early diagnosis. More efficient diagnostic tools are needed in clinical settings.

Metagenomic next-generation sequencing (mNGS) is notable for its broad-spectrum pathogen detection capability. Compared with conventional single-pathogen tests, mNGS demonstrates superior detection efficiency (19, 20). For CAP patients, mNGS demonstrated superior pathogen detection compared to conventional microbiological tests (21). Despite this, its demanding technical requirements and high cost restrict widespread clinical adoption.

Nasopharyngeal swab nucleic acid testing (NAAT) and serum IgM antibody assays are two accessible, non-invasive, and cost-effective methods for detecting *M. pneumoniae* in clinical practice. Despite the two methods having been widely used, and several studies have evaluated their performance in clinical practice, their diagnostic accuracy for MPP remains variable across studies. For instance, Kumar et al. reported that NAAT demonstrated a sensitivity of up to 100% and a specificity of 98.5%, whereas Chang et al. found a sensitivity and specificity of only 52.3% and 89.9%, respectively (22, 23). Similarly, for serum IgM antibody assays, Beersma and colleagues reported a sensitivity ranging from 35% to 77% and specificity between 49% and 100% (24). These discrepancies can be partially attributed to differences in reference standards (25–27). Prior studies used different reference standards (e.g., clinical diagnosis, serological testing, or culture confirmation) to judge the efficiency of the two non-invasive methods. However, to the best of our knowledge, no previous study has employed bronchoalveolar lavage fluid mNGS (BALF-mNGS)-a method proven to have exceptionally high pathogen detection capability in CAP- as the reference to evaluate the diagnostic performance of the two non-invasive tests in MPP. Therefore, we retrospectively evaluate the diagnostic accuracy (including sensitivity, specificity, etc.) of NAAT and serum IgM antibody assays for MPP, using a composite reference standard combining mNGS of BALF with clinician adjudication. We further assess non-inferiority of the two non-invasive methods compared to mNGS, with the goal of providing clinicians with evidence-based recommendations for selecting highly-accessible and high-performance diagnostic tools for MPP.

## 2 Materials and methods

### 2.1 Study design and setting

**Figure 1:**
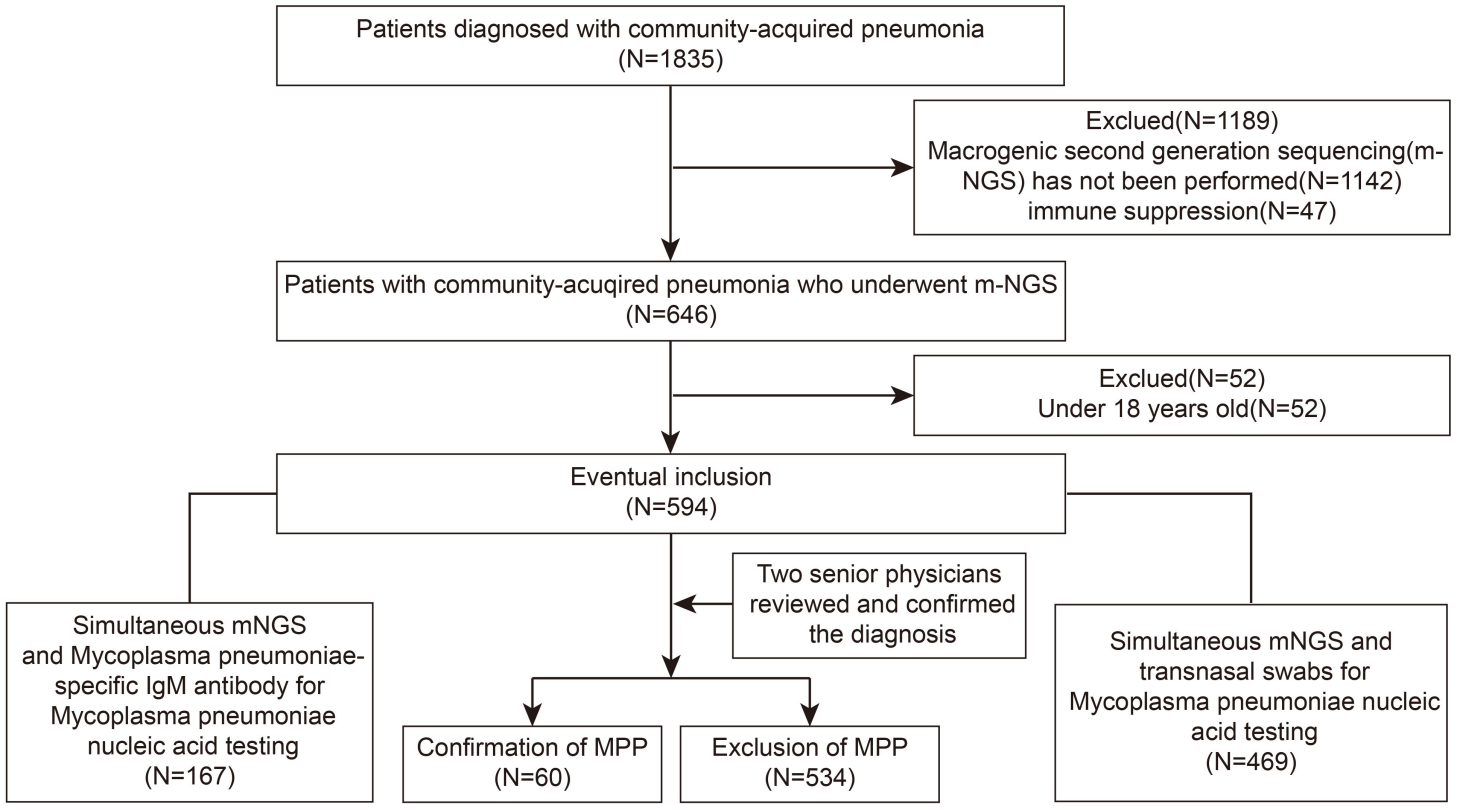
Flowchart of patient selection criteria. From January 1, 2024 to October 31, 2024, hospitalized patients with community-acquired pneumonia were screened. After exclusions (e.g., absence of BALF-mNGS or age <18 years), 594 patients were included in the analysis. Among them, 469 underwent NAAT and 167 underwent serum IgM antibody assays. Final diagnoses were adjudicated by two senior physicians.

We retrospectively reviewed the medical records of adult patients hospitalized with a final diagnosis of CAP at Zhongnan Hospital of Wuhan University between 1 January 2024 and 31 October 2024. The diagnostic criteria for CAP followed the guidelines issued by the Chinese Medical Association in 2016 (14). Patients were included if they underwent chest computed tomography (CT) confirming lesion localization and received at least one of two non-invasive tests, followed by fiberoptic bronchoscopy and bronchoalveolar lavage from the lesion site for mNGS. Diagnosis of MPP was based on criteria established by the World Health Organization (WHO) and the Centers for Disease Control and Prevention (CDC) for COVID-19 (28, 29), with the following requirements: (1) Laboratory testing (NAAT, serum IgM antibody assays or mNGS) indicated *Mycoplasma pneumoniae* infection; (2) Compatible clinical laboratory and radiological findings; and (3) Consensus confirmation by two board-certified senior physicians. Immunocompromised patients, including those receiving glucocorticoids, chemotherapy, or those with HIV infection, were excluded. The patient screening process is illustrated in **Figure 1**.

The study protocol was approved by the Medical Ethics Commission of Zhongnan Hospital of Wuhan University (Approval Number: 2025070K). As this is a retrospective study utilizing exclusively clinical data generated during routine diagnosis and treatment, the Ethics Committee waived the requirement for obtaining individual informed consent. All patient data were anonymized before analysis.

### 2.2 Samples and methods

Demographic characteristics, symptom duration before admission, clinical presentation, and results of ancillary tests performed within the first 24 hours of hospitalization were collected from patients’ records. The initial chest CT obtained after the onset of symptoms was included in the analysis. For patients who underwent outpatient testing before admission, the earliest post-onset results were recorded.

Serum *M. pneumoniae*-specific IgM antibodies were measured using a chemiluminescence immunoassay (CLIA) on the iFlash 3000 platform (YHLO Biotech Co., Ltd., Shenzhen, China), which applies a two-step indirect method. In the first incubation, *M. pneumoniae* IgM in the patient sample binds to *M. pneumoniae* antigens coated on paramagnetic particles, forming antigen–antibody complexes. These complexes are separated magnetically and washed to remove unbound substances. In the second incubation, acridinium-labeled mouse anti-human IgM binds to the complexes, generating antigen–antibody–secondary antibody complexes. Following a second wash, pre-trigger and trigger solutions are added, producing light signals measured as relative light units (RLUs). The RLU is proportional to the concentration of *M. pneumoniae* IgM in the sample, and results are interpreted by comparing the RLU to the assay’s predefined cutoff value. Test results were interpreted as positive when the cutoff index (COI) was ≥1.1, as per the manufacturer’s criteria. This assay is approved by the National Medical Products Administration of China (Registration No. 20173400758).

NAAT for *Mycoplasma pneumoniae* was performed on nasopharyngeal swab samples using a fluorescence probe-based real-time polymerase chain reaction (PCR) method. The assay targets a conserved region of the *M. pneumoniae* genome with specific primers and probes to ensure high analytical specificity, wherein the genomic target sequences and the sequences of the primers and probes are proprietary information of the manufacturer. Amplification and real-time detection of fluorescence signals were carried out on the AutoMolec 3000 system (Zhengzhou Antu Bioengineering Co., Ltd., Zhengzhou, China) using a commercially available PCR assay kit by the same company. This kit includes an internal control targeting the human globin gene to monitor the entire nucleic acid extraction and amplification process, thereby minimizing the occurrence of false-negative results. The presence of *M. pneumoniae* DNA in the sample was qualitatively determined by comparing the fluorescence signal to the assay’s predefined threshold cycle (Ct) value, as recommended by the manufacturer. According to the manufacturer’s instructions, a Ct value ≤ 36 was interpreted as a positive result. This assay, whose analytical sensitivity (limit of detection) and specificity were rigorously validated by the manufacturer, is approved by the National Medical Products Administration (NMPA) of China (Registration No. 20223401535).

### 2.3 Metagenomic next-generation sequencing of BALF samples

#### 2.3.1 Nucleic acid extraction

After mixing the BALF sample, 400 μL was added to a 2 ml centrifuge tube with 200 µL of 0.1 mm glass beads. Then the tubes were oscillated at 3000rpm/min for 5 minutes to break down the cell wall and release the nucleic acid. 300 μL supernatant was retained for DNA extraction by TIANamp Micro DNA Kit (Tiangen Biotech, China). At the same time, a 400 μL aliquot of Tris–EDTA buffer was processed as the negative control for DNA extraction (extraction control, ETC).

#### 2.3.2 Library preparation and sequencing

An in-house primer pool was used in this study. Amplification was performed individually in a 20 μL reaction system with 5 μL extracted DNA and 15 μL multiplex PCR mix, and using the following procedure: 1 cycle at 95°C for 3 min, 38 cycles at 95°C for 10 s, 55°C for 10 s, and 72°C for 10 s, followed by a final elongation step at 72°C for 5 min. The PCR products were mixed with equal quality for one sequencing library, which was prepared using the Nanopore Ligation Sequencing Kit (SQK-LSK109, Oxford Nanopore, Oxford, UK). The library was sequenced with an R9.4.1 flow cell (FLO-MIN106, Oxford Nanopore Technologies) by using Oxford Nanopore GridION X5.

#### 2.3.3 Bioinformatic analyses

The bioinformatics method and pathogen determination follow the previous study (30). FAST5 files containing raw Nanopore signal data were base called and converted to FASTQ format in real-time using Guppy (v3.3.0). High-quality data were obtained by removing exhibited low-quality reads (Q<7) and read with undesired length (<200 nt or >2000 nt). Adapter and barcode sequences were trimmed by using Porechop (v.0.2.4) from the high-quality data. After removing the adapter and barcode sequences, the following reads were compared with NT database (http://ftp.ncbi.nih.gov/blast/db) using BLASTn (v.2.9.0+). The taxon of the reads assigned to the same species was based on a consensus sequence was made by Medaka (v.0.10.1) from the reads assigned to the same species and compared with the reference databases. The criteria used to select these pathogens as follows: (1) Filtering out closely related microorganisms (31). (2) Filtering out contaminants from negative controls (32). The final report for each sample was discussed individually with a clinical microbiology specialist and clinicians responsible for each patient.

### 2.4 Statistical analysis

Statistical analyses were conducted using IBM SPSS Statistics 25. For normally distributed data, the Student t-test was used to compare differences between the two groups. For non-normally distributed data, the Mann-Whitney U test was used for analysis. Pearson’s chi-square test or Fisher’s exact test were employed to assess differences in categorical variables between the two groups. McNemar’s test was used to compare the sensitivity of two non-invasive diagnostic methods (serum IgM antibody assays and NAAT) against mNGS, with statistical significance set at *P* < 0.05. A non-inferiority margin of Δ = 0.10 was applied to determine whether the difference in sensitivity fell within the non-inferiority threshold. Non-inferiority testing for sensitivity was performed by calculating the difference in sensitivity between NAAT/IgM and mNGS, along with 95% confidence intervals (CI). If the lower bound of the CI was greater than –10%, the test was considered non-inferior. The 95% CI of the paired differences were calculated using the Newcombe method.

### 2.5 Combined diagnostic strategy evaluation

To assess whether combining the two non-invasive diagnostic tests would improve detection sensitivity, we constructed a composite indicator in which a positive result from either NAAT or serum IgM antibody assays was defined as a positive outcome. This combination aimed to optimize early identification of MPP using less-invasive methods.

## 3 Results

### 3.1 Patient characteristics

A total of 594 patients were enrolled. Among them, 60 (10.10%) were diagnosed with MPP, while 59 cases were confirmed by positive mNGS results supplemented by clinical diagnosis; 1 case was confirmed by positive NAAT supplemented by clinical diagnosis. Among the 594 patients, 469 completed nucleic acid testing, with 43 positive and 426 negative results. Additionally, 167 patients underwent IgM antibody testing, with 17 positive and 150 negative results. Compared with patients with other pneumonia, those with MPP were significantly younger (35.00 years [27.00–49.75] vs. 64.00 years [54.00–72.00], *P* < 0.001). In terms of imaging findings, MPP was more frequently characterized by unilateral lung involvement (38.33% vs. 65.73%, *P* < 0.001). Baseline patient characteristics are summarized in **Table 1**.

**Table 1:** Baseline characteristics of study participants.

Characteristics	Patients diagnosed with MPP (n=60), Median (IQR)	Patients diagnosed with other pneumonia (n=534), Median (IQR)	P-Value
Age (years)	35.00 (27.00-49.75)	64.00 (54.00–72.00)	<0.001
Gender, n(%)			0.02
Males, n (%)	27(45.00)	321 (60.11)	
Females, n (%)	33(55.00)	213 (39.89)	
Chest CT findings, n (%)			
Lesions involving both lungs, n (%)	23(38.33)	351(65.73)	<0.001

MPP, *M. pneumoniae* pneumonia; Data are presented as Median (IQR) or n (%). P-values were calculated using the Mann–Whitney U test for continuous variables and the χ² test for categorical variables.

### 3.2 Testing efficiency

Among 469 patients who underwent NAAT for *M. pneumoniae*, 58 (12.4%) were ultimately diagnosed with MPP. NAAT demonstrated a sensitivity of 74.1% and specificity of 99.3% compared with composite diagnostic criteria. Among the 167 patients who underwent serum IgM antibody assays, sensitivity was 23.6% and specificity was 98.0%. In comparison, mNGS of BAL fluid exhibited a sensitivity of 98.3% and specificity of 100%, serving as the reference standard. The concrete results are presented in **Table 2**.

**Table 2:** Diagnostic performance of PCR, IgM, and mNGS in detecting MPP.

Diagnostic method	Sensitivity (%)	Specificity (%)	PPV	NPV	+LR	−LR
NAAT	74.1	99.3	93.5	96.5	105.9	0.26
Serum IgM antibody assays	23.6	98.0	57.1	91.9	11.8	0.78
mNGS (BAL fluid)	98.3	100.0	100.0	99.8	∞	0.02

MPP, *M. pneumoniae* pneumonia; PCR, Nasopharyngeal swab nucleic acid testing; mNGS, Metagenomic next-generation sequencing; PPV, Positive Predictive Value; NPV, Negative Predictive Value; LR, Likelihood Ratio; BAL, Bronchoalveolar Lavage; ∞=mathematically undefined (perfect specificity).

### 3.3 Non-inferiority analyses

Among patients with confirmed MPP who completed both NAAT and mNGS (n = 58), the sensitivity of NAAT and mNGS were 74.1% (43/58) and 98.3% (57/58). The absolute difference in sensitivity was -24.2% (95% CI: -36.1 to -12.1%). Similarly, a total of 17 patients with confirmed MPP underwent both serum IgM antibody assays and BALF mNGS testing. The sensitivity of IgM antibody was 23.6% (4/17). Compared with mNGS, the sensitivity difference was –76.5% (95% CI: –96.6% to –56.3%). Neither serum IgM antibody assays nor NAAT met the prespecified non-inferiority margin of -10%. McNemar’s test indicated statistically significant differences in sensitivity between both serum IgM antibody assays and mNGS (*p*<0.01), and NAAT and mNGS (*p*<0.01). The concrete analysis data are summarized in **Table 3**.

**Table 3:** Non-inferiority analysis and McNemar’s test comparing sensitivity of IgM antibody and PCR with mNGS in confirmed MPP.

Comparison	Sample size (n)	IgM/PCR test(+)	mNGS test(+)	Sensitivity difference (%)	95% confidence interval (%)	Non-inferiority conclusion (δ = -10%)	McNemar’s test p-value
IgM antibody vs mNGS	17	4	17	-76.5	-96.6 to -56.3	Not Non-Inferior	< 0.01
PCR vs mNGS	58	43	57	-24.1	-36.1 to -12.1	Not Non-Inferior	< 0.01

MPP, *M. pneumoniae* pneumonia; PCR, Nasopharyngeal swab nucleic acid testing; mNGS, Metagenomic next-generation sequencing; IgM, Serum IgM assays; Non-inferiority was defined as the lower bound of the 95% confidence interval for the sensitivity difference being above -10%.

### 3.4 Evaluation of the combined diagnostic strategy

To evaluate the diagnostic performance of the composite indicator (defined as positive if either serum IgM antibody assays or NAAT was positive), a 2×2 contingency table was constructed using the final clinical diagnosis as the reference standard. Among the 15 patients ultimately diagnosed with MPP, 13 tested positive using the composite method, resulting in a sensitivity of 86.7% (13/15). All 27 patients without MPP tested negative, yielding a specificity of 100% (27/27). The concrete diagnostic indicators are summarized in **Table 4**.

**Table 4:** Diagnostic performance of the combined diagnostic strategy (IgM or PCR positive) in confirmed MPP.

Diagnostic method	Sensitivity (%)	Specificity (%)	PPV	NPV	+LR	−LR
Combined Diagnostic Strategy	86.7	100	100	93.1	∞	0.13

MPP, *M. pneumoniae* pneumonia; PPV, Positive Predictive Value; NPV, Negative Predictive Value; LR, Likelihood Ratio; ∞=mathematically undefined (perfect specificity).

To further assess whether the composite indicator was non-inferior to mNGS in terms of sensitivity for identifying MPP, a non-inferiority analysis was conducted among the 15 patients with confirmed MPP who had completed both tests. The sensitivity of the composite test was 86.7% (13/15), compared to 100% (15/15) for mNGS. The sensitivity difference was –13.3% (95% CI: –30.5% to +3.9%). Since the lower bound of the CI (–33.6%) did not exceed the predefined non-inferiority margin of –10%, the composite indicator could not be considered non-inferior to mNGS in this cohort.

## 4 Discussion

In this retrospective study, we evaluated the diagnostic performance (sensitivity, specificity,etc.) of two widely used non-invasive tests—NAAT and serum IgM antibody assays in the diagnosis of MPP—against a reference standard based on BALF-mNGS. Our findings showed that NAAT detected approximately three quarters of confirmed cases (sensitivity 74.1%) and maintained a specificity above 95%, whereas IgM antibody assays identified a smaller proportion of true cases (sensitivity 23.6%) but demonstrated a similarly high specificity. These results indicate that both methods are reliable for confirming MPP when positive, but their limited sensitivity means that negative results cannot reliably exclude infection in clinically suspected patients. Furthermore, the non-inferiority tests demonstrated that neither non-invasive test alone, nor their combination, reached the predefined non-inferiority margin against BALF-mNGS in terms of sensitivity. This confirms that mNGS remains the most definitive diagnostic tool in the current clinical setting.

In current clinical practice, NAAT is widely utilized as a supplementary diagnostic tool for various pathogens, including SARS-CoV-2 and influenza A/B viruses, and has demonstrated high diagnostic efficacy (33, 34). However, few studies have investigated its diagnostic accuracy in MPP (35). Our results showed that NAAT had a specificity of 99.3% and a positive predictive value of 93.5%. These findings suggest that a positive result from NAAT for *M. pneumoniae* can provide strong evidence for diagnosing MPP, thereby supporting the initiation of targeted therapy. Furthermore, a recent study demonstrated that NAAT can substantially improve early detection of MPP, with positive rates exceeding 70% (36), which was consistent with our findings.

By contrast, the sensitivity of NAAT was 74.1%, indicating that a negative result cannot reliably exclude MPP. Several factors may account for this finding. First, the positive rates of nucleic acid testing are associated with differences in sampling technique. Inadequate sampling may fail to collect sufficient secretions from the site of infection, leading to false-negative results. Second, in MPP patients, low concentrations of *M. pneumoniae* in the upper respiratory tract may also result in false-negative outcomes for nucleic acid testing (37).

Regarding serum IgM antibody assays, our study found excellent specificity (98.0%), indicating that a positive serological result can serve as supplementary evidence for diagnosing MPP. However, its sensitivity was only 23.6%, suggesting that over 75% of patients with MPP would not be detected by this method. A comparative study by Qu et al. involving patients with CAP reported similar findings, with low sensitivity for serum IgM antibody assays, but with high specificity (36). Several main factors may account for the low sensitivity observed. First, multiple studies have shown that *M. pneumoniae-*specific IgM antibodies typically appear around seven days after symptom onset, peak at three to six weeks, and persist for two to six months (37, 38). In our study, some patients presented for medical attention within seven days of symptom onset, which may have contributed to the relatively low sensitivity of serum IgM antibody assays. Second, specific populations, such as elderly patients, have weaker humoral immune responses, which can affect IgM antibody production (39). Except for the two factors mentioned above, several studies have suggested that the sensitivity of serum IgM antibody assays may vary to some extent depending on the methodology used, the choice of diagnostic cut-off values (37, 40). Also, compared to previous studies, our study adopted a composite reference incorporating BALF-based mNGS, which has demonstrated higher pathogen detection sensitivity (21, 41). As a result, mNGS may have identified cases at earlier stages of infection, when specific IgM antibodies were not yet detectable, thereby reducing the apparent sensitivity of the serological test. Therefore, for patients with suspected MPP, repeating IgM testing after 7 days of symptom onset or utilizing higher-sensitivity assays may improve pathogen detection rates, thereby supporting diagnostic decision-making (25, 37, 38).

In order to optimize diagnostic sensitivity while minimizing unnecessary costs and procedural risks, we integrated two non-invasive assays into a composite indicator and evaluated its non-inferiority against BALF-mNGS in sensitivity. However, the composite indicator failed to demonstrate statistical non-inferiority to the latter. Therefore, in cases of poor response to empirical therapy, chronic respiratory comorbidities, or suspected severe pneumonia, mNGS is recommended for pathogen identification to guide targeted antibiotic therapy (20, 42, 43).

One strength of this study lies in its real-world, retrospective design, which enables the evaluation of diagnostic performance in actual hospital settings. This approach reflects how diagnostic tools are applied in clinical practice, where patients present at variable stages of illness and tests are selected based on routine availability. Both NAAT and serum IgM antibody assays are widely used, accessible, and demonstrat high specificity in our study, supporting their continued role as practical adjunctive tools in the diagnostic workup of MPP, especially in resource-limited or outpatient settings.

This study has several limitations. First, this study included a limited subcohort of 167 patients who underwent serum IgM antibody assays, and the statistical power of these analyses requires further validation in larger cohorts. Second, as a single-center retrospective study, our findings may be influenced by local patient demographics. Larger, multicenter prospective cohorts are needed to confirm generalizability across diverse healthcare settings.

## 5 Conclusion

Nasopharyngeal swab nucleic acid testing demonstrates excellent specificity and acceptable sensitivity, supporting its use as a reliable, non-invasive tool for the early diagnosis of suspected MPP. In contrast, although serum IgM antibody assays exhibit high specificity, their limited sensitivity restricts their value in early diagnosis of MPP. The non-inferiority analysis revealed that neither non-invasive testing modality demonstrated sensitivity comparable to mNGS, failing to meet the pre-specified non-inferiority margin. For MPP patients, mNGS remains the most effective diagnostic modality currently.

## Data Availability

All data analyzed during this study are included in this published article and its supplementary information files. All data were fully anonymized prior to access.
